# Recent Trend of Laboratory Tests in Common Gastrointestinal Tract Disorders

**DOI:** 10.3390/diagnostics15232998

**Published:** 2025-11-26

**Authors:** Terence A. Agbor, Waliul I. Khan

**Affiliations:** 1 Department of Pathology and Molecular Medicine, McMaster University, Hamilton, ON L8S 4L8, Canada; terence.agbor@lifelabs.com; 2 LifeLabs, Medical-Scientific Department, 100 International Blvd., Toronto, ON M9W 6J6, Canada; 3 Hamilton Regional Laboratory Medicine Program, Hamilton Health Sciences, Hamilton, ON L8S 4L8, Canada; 4 Farncombe Family Digestive Health Research Institute, McMaster University, Hamilton, ON L8S 4L8, Canada

**Keywords:** laboratory diagnosis, GI disorders, *Helicobacter pylori*, IBD, IBS, celiac disease, colon cancer

## Abstract

The gastrointestinal (GI) tract is a complex organ system affected by multiple disorders with diverse etiologies ranging from infections to immune dysfunction disorders and cancers. Various GI disorders, such as *Helicobacter pylori* infection, inflammatory bowel disease (IBD), celiac disease, irritable bowel syndrome (IBS), and colon cancer, are common and cause significant morbidity, mortality, and healthcare costs. These disorders present with overlapping signs and symptoms, warranting the need for accurate laboratory diagnostic tests for appropriate treatment implementation and treatment monitoring. The gold standard confirmatory diagnostic test for most GI disorders is endoscopy and biopsy for histological analysis. Biomarkers in blood and stool are also routinely used either as first-line screening tests or for treatment monitoring in many GI disorders. This review summarizes common GI disorders along with related currently used clinical laboratory tests in screening, diagnosis, and monitoring of these diseases, outlining the methodology, utilization, advantages, and limitations of these tests. We also highlight the effectiveness of each test as well as the professional recommendations and clinical guidelines for their use where available. Finally, we shed some light on potential future tests and biomarkers that aid in diagnosing GI disorders and how these biomarkers can be used in conjunction to complement the current tests. Some of the potential future biomarkers discussed include the differential expression of gut microbiota and their respective metabolites, as well as cytokines, as potential tests that can be used to diagnose diseases, distinguish between disease subtypes, predict disease severity and occurrence, and optimize treatment decisions. Comprehending the effectiveness of various methodologies for laboratory diagnosis of GI disorders is crucial for health care personnel, including clinical laboratory professionals and clinicians, regarding testing options, test utilization, and interpretations of results. Insights into future tests in GI diseases in the context of microbiomes, metabolites, and immune mediators based on advanced technology are also important in their appropriate clinical utilization.

## 1. Introduction

The gastrointestinal (GI) tract is a complex organ system running from the mouth to the anus that performs a wide range of critical physiological functions, which include digestion, absorption, metabolism of ingested food, water balance, and waste elimination. Further to these functions, which are maintained by the various organs discretely, there is now emerging evidence that supports the importance of the microbiome, composed of a collection of bacteria, fungi, viruses, and archaea, in the physiology and pathophysiology of the GI tract [1]. The global burden of many GI diseases has been on the rise over the last few decades [2]. According to a study, in 2019, globally, there were 7.32 billion incidences and 2.86 billion prevalent cases of GI diseases, resulting in 8 million deaths [3]. The prevalence was significantly influenced by geography and socioeconomic status. In addition, GI diseases account for substantial health care use and expenditures [4]. Most GI diseases result in structural and physiological changes in the gut due to infection or injury, which culminates in the signs and symptoms of GI disorders, including but not limited to disorders of gut motility, such as irritable bowel syndrome (IBS), disorders of secretory dysfunction, such as peptic ulcer disease from *Helicobacter pylori* infection, malabsorption and immune-mediated disorders, including celiac disease and inflammatory bowel diseases (IBD), as well as neoplastic disorders, notably, colorectal cancer.

The common clinical symptoms of GI diseases include abdominal pain, heartburn, nausea, vomiting, bloating, diarrhea, constipation, and GI bleeding. Many GI diseases present with overlapping symptoms, warranting the need for the involvement and collaboration of a multidisciplinary healthcare team, including clinicians, laboratory personnel, radiologists, and surgeons. Appropriate measures for proper screening, diagnosis, and therapeutic monitoring are key to reducing the high burden of GI diseases. Recently, there have been significant advances in the diagnosis of GI diseases by implementing immunological and molecular laboratory tests, radiological approaches, and GI endoscopy with artificial intelligence. The purpose of this review is to discuss the recent trends in laboratory tests in common GI disorders, as well as advances in this area for future novel tests.

## 2. Common GI Disorders and Related Laboratory Tests

### 2.1. Helicobacter pylori Infection

*Helicobacter pylori* is a Gram-negative bacterium capable of colonizing the acid-rich gastric mucosa of the stomach. It is the most common causative agent of gastric infections, occurring in 40–60% of the general population worldwide [5,6,7,8,9] with a variable geographical prevalence, and is the leading cause of peptic ulcer disease, chronic and atrophic gastritis, gastric lymphoma, and gastric carcinoma [10]. There are both invasive and non-invasive methods for diagnosing *H. pylori* infection. The gold standard for the diagnosis of *H. pylori* infection is endoscopy and biopsy of the gastric mucosa for histological examination and detection of urease, which converts urea to ammonia and carbon dioxide (CO_2_)—an inherent physiological property of the bacteria [11]. For histological examination, various immunohistochemical stains and peptide nucleic acid fluorescent in situ hybridization (PNA-FISH) highly specific (100%) and sensitive (97%) are used [5,12,13].

The rapid urease test for biopsy samples is cost-effective, rapid, easy to perform, and specific. Briefly, a tissue sample collected after a biopsy is transferred into a urea solution containing a pH indicator [14,15]. In the presence of *H. pylori*, the bacterial urease will convert the urea to ammonia and CO_2_, resulting in a colour change of the solution due to the alkalinization of the solution. As will be discussed later, endoscopy is an invasive procedure with many limitations. Invasive methods also include culturing *H. pylori* from a gastric biopsy specimen, which allows for antimicrobial sensitivity testing [15,16,17].

Non-invasive laboratory diagnostic tests for *H pylori* include the urea breath test, the *H. pylori* stool antigen test, serological tests (IgM and IgG), and molecular tests (for detection of *H. pylori* nucleic acid in fecal samples).

#### 2.1.1. Urea Breath Test

The urea breath (UBT) is considered the gold standard non-invasive test for the detection of *H. pylori* and utilizes the bacteria’s urease enzyme, which breaks down urea into ammonia and CO_2_ [11]. The CO_2_ released is then measured in the patient’s breath samples. Briefly, the patient is given a drink (solution) containing urea isotopically labelled with carbon-13 (^13^C—non-radioactive). In the presence of *H pylori* in the stomach, the urea is hydrolyzed by the bacteria’s urease enzyme, releasing CO_2_, which diffuses across the gut mucosa into circulation and is exhaled. Breath samples are collected between 20 and 40 min after consumption of the drink, and the change in the proportion of C-13 in the exhaled air compared to a control is determined by isotope ratio mass spectrometry. Alternative methods for determining 13C-labelled carbon dioxide activity include infrared spectroscopy and laser-assisted ratio analysis. The UBT is relatively accurate, with sensitivity and specificity of ≥95%, respectively [5,18]. Patients are required to stay off proton pump inhibitors (PPIs), antibiotics, or bismuth compounds, which suppress *H. pylori* and urease activity, for 2–4 weeks before testing. Another common cause of false negatives is bleeding, and it is recommended that the UBT be delayed after recovery from bleeding to optimize test accuracy [19]. The major limitations of the UBT include long patient wait times, logistical challenges, including cost, specialized training, and equipment (isotope ratio mass spectrometry), patient preparation and cooperation, and compliance in children under 5 years.

#### 2.1.2. *H. pylori* Stool Antigen Test (HpSAT)

An alternative to the UBT is the *H. pylori* stool antigen test (HpSAT). HpSAT is a fecal immunological test used for the detection of the *H. pylori* antigen in stool samples of infected patients. The HpSAT is used to diagnose active infection and thus is subject to fewer false positives. The test is easy to perform and available on many automated laboratory platforms. Currently available assays are based on the enzyme-linked immunosorbent assay (ELISA), immunochromatographic assay, and chemiluminescence immunoassay (CLIA) methodologies using monoclonal antibodies. Polyclonal antibody-based assays are also available but not recommended due to decreased specificity. The test is recommended for diagnosing *H. pylori* infection in pediatric patients and in patients where the UBT cannot be performed, such as patients with asthma or achlorhydria and after gastrectomy. The accuracy of the HpSAT is comparable to the UBT, with sensitivity and specificity values of monoclonal antibody-based assays of 93% and 96% or higher, respectively [5,20,21,22]. Like the UBT, patients are required to stay off proton pump inhibitors (PPIs), histamine H2 inhibitors, antibiotics, and bismuth compounds for 2–4 weeks prior to sample collection for testing [23]. The transport temperature and the duration of transporting fecal samples to the central laboratory for testing may also affect the accuracy of the test results.

#### 2.1.3. *H. pylori* Serology

Serological assays for detecting antibodies against *H. pylori* IgA, IgM, and IgG are also available [14,24]. In acute infection, IgM anti-*H. pylori* antibodies can be detected, while IgA and IgG are detectable in chronic infections. Despite the availability of assays for detecting anti-IgA and anti-IgM–*H. pylori* antibodies, these tests are associated with high rates of false positive results [25]. The detection of IgG is a reliable screening test for *H. pylori* infection, with sensitivity and specificity values of 99% and 96%, respectively [18,26,27]. However, antibody testing is not reliable to confirm active infection, as IgG persists in circulation even after successful treatment and eradication of the bacteria. An important clinical use of IgG serology is the assessment of peptic ulcer disease [28,29,30]

#### 2.1.4. *H. pylori* Molecular Testing

Molecular testing, including the polymerase chain reaction (PCR-based methodologies), has recently emerged for the non-invasive detection of *H. pylori* infections in fecal samples. One such method combines the detection of *H. pylori* DNA in fecal samples and point mutations on the 23S ribosomal RNA gene of *H. pylori*, which are associated with Clarithromycin antibiotic resistance [21,31]. This method has very high analytical sensitivity with the added benefit of being able to guide antibiotic therapy. The clinical utility, advantages, and limitations of each *H. pylori* test are summarized in Table 1.

Several non-invasive laboratory tests are currently available for the detection and assessment of treatment effectiveness for *H. pylori* infection. The UBT and HpSAT have similar sensitivity and specificity, while molecular testing can also guide antibiotic therapy. The test of choice should be determined by available resources and test complexity.

### 2.2. Celiac Disease

Celiac disease is a chronic, autoimmune, and inflammatory disease of the small intestine caused by sensitivity to gluten (gliadin) and other prolamins present in wheat, barley, and rye in genetically susceptible individuals, affecting ~1% of the world’s population [32,33,34,35]. Genetically, the disease has a close association with the human leukocyte antigen (HLA) DR3-DQ2 and/or DR4-DQ8 gene locus, with over 99% of individuals with celiac disease having the HLA DR3-DQ2 and/or DR4-DQ8 allele, compared with 30–40% of the general population [36,37]. Pathophysiologically, following the consumption of gluten-rich foods, gluten is deamidated in the intestinal mucosa (lamina propria) by tissue transglutaminase (tTG), which increases its binding affinity to HLA-DQ2 and HLA-DQ8 [38,39]. The HLA–gluten complex activates CD4+ T cells, leading to the release of pro-inflammatory cytokines and chemokines and promoting the infiltration of cytotoxic CD8+ T cells into the lamina propria, which culminates in mucosal inflammation. Celiac disease presents both GI and extraintestinal symptoms. The classic signs and symptoms of celiac disease are characteristic of malabsorption and include diarrhea, abdominal bloating, steatorrhea and flatulence, chronic fatigue, poor growth and failure to thrive, nutrient deficiencies, anemia, and short stature. Delayed puberty, dental enamel hypoplasia, reduced bone density, oral ulcers, liver and biliary disease, and dermatitis herpetiformis are also common.

Most national and international guidelines recommend the use of serology as the first-line testing for patients with the signs and symptoms of celiac disease, followed by upper duodenal biopsies and histology [40,41]. Duodenal biopsies followed by histology are considered the gold standard for the diagnosis of celiac disease [42,43]. Using this approach, the measurement of anti-tTG IgA in serum samples is the recommended first-line serology test with concomitant measurements of total IgA to rule out IgA deficiency. It is imperative that patients consume their normal diet (i.e., gluten-containing diet) prior to testing due to the normalization of serology when patients switch to a gluten-free diet. The tTG-IgA test has excellent performance, with a sensitivity and specificity of >90% and a positive predictive value close to 100% when the levels are >10× the upper reference limit (URL) of normal [42,43,44]. In patients with IgA deficiency, IgG-based serological testing for tTG (anti-tTG IgG), deamidated gliadin peptide (DGP IgG), or anti-endomysial antibodies IgG (EMA-IgG) should be performed [41,43,45]. In adults with a positive anti-tTG IgA (or positive anti-tTG IgG or DGP-IgG, in the case of IgA deficiency), confirmatory testing by duodenal biopsy and histology should be performed. The classic histological picture of a celiac patient shows villous atrophy, crypt hyperplasia, and intraepithelial lymphocytosis, as classified by Marsh and later modified by Oberhuber et al. [46,47,48]. In both adults and children, a positive anti-tTG and normal biopsy is consistent with potential celiac disease, with an abnormal biopsy (Marsh 1–3 criteria) diagnostic of celiac disease [41]. In children, celiac disease can be diagnosed based on serological testing alone without the need for a biopsy. Elevated anti-tTG IgA results >10× the URL of normal with a positive EMA in a second blood sample can be reliably used to diagnose celiac disease. EMA testing is performed in specialized laboratories by indirect immunofluorescence on tissue substrates, requiring an expert and skilled technologist to interpret results, making it time-consuming and labour-intensive, as well as subjective in results interpretation. In children with tTG-IgA levels <10× the URL of normal, duodenal biopsies are recommended [45]. HLA-DQ2/DQ8 testing is reserved for individuals with high clinical suspicion of celiac disease, but who are on a gluten-free diet, since serology and histology can normalize on a gluten-free diet.

**Figure 1:**
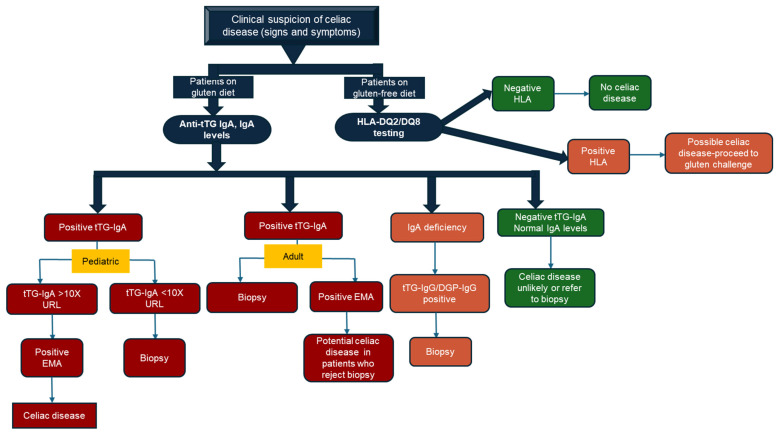
Modified celiac disease screening and diagnostic algorithm based on serology testing in patients on a normal gluten diet. In children with signs and symptoms of celiac disease, high levels of tTG IgA (>10× URL) with a positive EMA in a second blood sample is diagnostic of celiac disease. Children with positive tTG-IgA below 10× URL are referred for biopsy. In adults, patients with positive tTG-IgA should undergo a biopsy to confirm the diagnosis of celiac disease. In patients with IgA deficiency, tTG-IgG or DGP-IgG should be performed. In patients on a gluten-free diet, HLA-DQ2/DQ8 testing can be performed. DGP, deamidated gliadin peptide; EMA, endomysial antibody; HLA, human leukocyte antigen; IgA, immunoglobulin A; IgG, immunoglobulin G; anti-tTG, tissue transglutaminase antibody, URL, upper reference level (adapted from [42]).

Figure 1 summarizes the algorithm for celiac testing, as recommended by most international guidelines. Some of the limitations of using serology alone to diagnose celiac disease include differences in assay performance between different manufacturers, use of different diagnostic cut-offs and reference intervals, medications, such as immunosuppressants, infections that result in false negative results, and, finally, uncertainty associated with not using the gold standard test—biopsy and histology [49,50,51]. The clinical utility of each celiac serologic test is summarized in Table 2. In summary, the diagnostic algorithm for celiac disease begins with the determination of tTG-IgA and total IgA in patients on a normal gluten diet, followed by duodenal biopsy in adults (or biopsy-free diagnosis in children with tTG-IgA >10× URL and positive EMA), with IgG-based tests used in IgA deficiency and HLA-DQ2/DQ8 testing reserved for patients on a gluten-free diet.

### 2.3. Inflammatory Bowel Disease

Inflammatory bowel disease (IBD), Crohn’s disease (CD), and ulcerative colitis (UC) are serious chronic inflammatory conditions of the human bowel that currently affect approximately 1–2 million people in the US and Canada and several million worldwide [52,53]. Although the exact etiology of IBD is not known, studies have provided evidence that a dysregulated immune response, genetic factors, the gut microbiota, and environmental factors contribute to the pathogenesis of IBD [54,55,56]. Mucosal inflammatory activity manifests as symptoms of pain, diarrhea, and bleeding with or without features of systemic inflammation. In UC, inflammation is limited to the colon: it begins in the rectum and spreads continuously proximally. In CD, any part of the GI tract may be involved, most commonly the terminal ileum or the perianal region. Inflammation in CD is non-continuous and commonly associated with complications, such as strictures, abscesses, and fistulas [56,57,58]. Histologically, in UC, the inflammatory changes are limited to the mucosa and submucosa, with cryptitis and crypt abscesses [59]. The gold standard for the diagnosis and characterization of IBD is direct assessment of ongoing mucosal inflammation by endoscopy [60,61,62]. This procedure is invasive, expensive, and associated with significant patient preparation, long waiting times, and discomfort while carrying a small but significant risk of complications [63,64]. Laboratory testing using blood and fecal biomarkers is routinely used to guide diagnosis and monitor therapy and recurrence.

#### 2.3.1. Fecal Calprotectin

Recently, determining calprotectin levels in stool samples has emerged as a useful biomarker for IBD. Calprotectin is a 36 kDa heterodimeric calcium- and zinc-binding protein complex; it represents ~5% of the total protein in neutrophils and accounts for ~40–60% of the cytosolic proteins of neutrophils and, to a lesser extent, monocytes and macrophages. Calprotectin has become a mainstay biomarker for screening, diagnosis, and monitoring patients with IBD in fecal samples [65]. The relatively high amount of transepithelial neutrophil migration and turnover in the gut lumen during inflammatory disorders of the gut provides an opportunity to utilize calprotectin in stool (fecal calprotectin—FC) as a biomarker of IBD. FC is considered a surrogate non-invasive marker of the infiltration of neutrophils in the intestinal mucosa, and an increase in FC levels is associated with intestinal inflammation [64,66,67,68]. It plays an increasingly crucial role in the management of IBD patients, with levels closely correlated with endoscopic and histological disease activity. It is also used to distinguish IBD from irritable bowel syndrome (IBS), assess disease activity and response to therapy, and predict disease recurrence [64,69]. Several meta-analyses and systematic reviews have indicated an overall sensitivity of 80–98% and a specificity of 68–96%, with an FC threshold ranging from 30 to 100 µg/g in adults, where most cite 50 µg/g [66,69,70].

**Figure 2:**
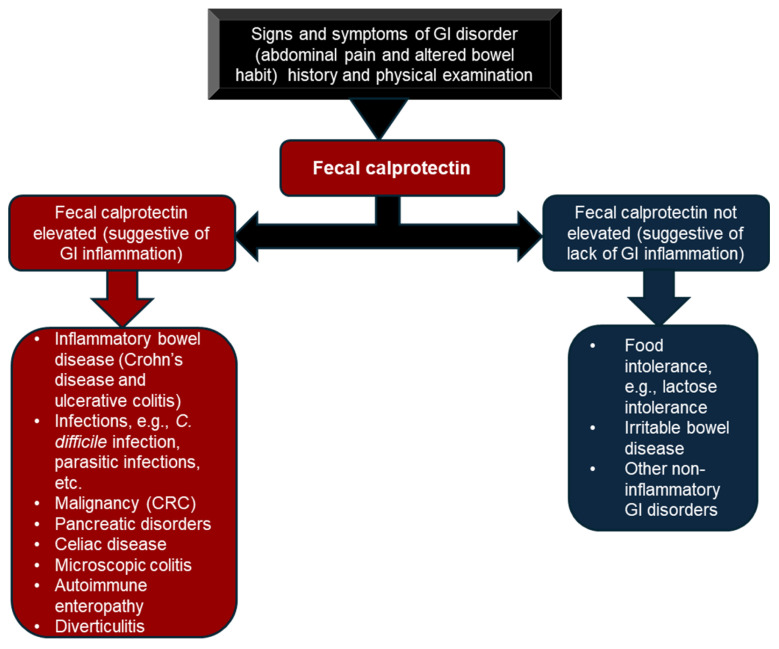
Fecal elevation in GI disorders. Fecal calprotectin is elevated in many inflammatory disorders of the GI tract. While calprotectin is specific to inflammation, it is not specific to IBD. Elevation of calprotectin can be observed in other GI inflammatory disorders, such as bacterial and parasitic infections, GI cancers, such as CRC, celiac disease, and microscopic colitis. Fecal calprotectin is used to distinguish inflammatory from non-inflammatory disorders of the GI tract, such as irritable bowel syndrome.

While calprotectin may be specific to inflammation, it can be elevated in other inflammatory and infectious conditions of the GI tract, including autoimmune enteropathy, diverticulitis, bacterial dysentery, intestinal polyps, and colon cancer, making it less specific to IBD (Figure 2).

The main clinical utilities of FC in IBD patients include assessing inflammation and neutrophil activity in the gut, i.e., disease recurrence and active inflammation, distinguishing IBD from irritable bowel syndrome (IBS), monitoring mucosal inflammation, and predicting relapse as well as response to therapy [71,72,73] (Table 3).

An emerging utility of FC is as a triaging test in patients with suspected disease for specialist referral and endoscopy. Patients with elevated FC (active disease) may directly proceed to endoscopy, which can lead to early disease diagnosis and treatment initiation. Although the normal range for FC (≤50 µg/g) is defined, there is no validated, precise cut-off in FC assays to define active IBD and/or remission. This also applies to predicting relapse, assessing mucosal healing, and monitoring response to treatment. Recently, an FC algorithm for the detection of IBD was proposed [74]. According to this algorithm, (1) <lower cut-off: <100 µg/g (inflammation highly unlikely; not suggestive of IBD; invasive testing can be deferred); (2) grey zone: >100 + <250 µg/g (suggestive of low-grade inflammation; interpret with caution; consider repeat testing); and (3) >higher limit: >250 µg/g (significant inflammation present; IBD likely; perform ileocolonoscopy). Another study suggested an FC concentration of >400 µg/g to rule in IBD and proceed to endoscopy [69,75,76]. An important consideration is the variability in calprotectin concentrations across different age groups, with high concentrations observed in pediatric patients. Day-to-day variability in FC and single-day intra-individual variation in patients are also observed [77,78].

#### 2.3.2. Fecal Lactoferrin

Lactoferrin is an ~80 kDa iron-binding glycoprotein present in mucosal membranes and found in various secretions, including breast milk, saliva, tears, and nasal secretions. It is a component of secondary granules in neutrophils and possesses antimicrobial activity [79]. Infiltration of neutrophils into the gut lumen during intestinal inflammation, which is characteristic of IBD, results in an increase in lactoferrin in feces. Like FC, fecal lactoferrin (FL) shows similar clinical sensitivity and specificity to gut inflammation [62,79,80]. Thus, it is not necessary to use both assays in the same institution.

Another emerging biomarker of gut inflammation is fecal calgranulin C. Calgranulin C (f-S100A12) is a calcium-binding protein exclusively expressed by granulocytes. It plays an important role in the pathogenesis of IBD and is more specific to intestinal inflammation [81,82]. Several studies have shown a significant correlation between elevated f-S100A12 and intestinal inflammation. Fecal S100A12 levels were significantly elevated in patients with either active or inactive IBD and bacterial gastroenteritis but not in patients with IBS or viral gastroenteritis, compared to healthy controls [81]. The use of this biomarker in the clinic is limited at this time, but it may be of potential significance in the future.

#### 2.3.3. C-Reactive Protein (CRP)

CRP is a pentameric acute-phase protein produced principally by hepatocytes in response to inflammatory cytokines, such as IL-6 [82,83]. CRP has a relatively short half-life (~19 h), making it useful in the assessment of the onset and resolution of inflammation. In IBD, CRP has been shown to correlate with disease activity in pediatrics, showing moderate correlation with endoscopic findings and active mucosal inflammation, with a rise in CRP levels dependent on the extent of inflammation [84,85]. The pooled sensitivity and specificity of CRP for detecting endoscopic activity in IBD patients have been reported in the range of 49% and 92%, respectively [85]. Elevated CRP levels have also been shown to correlate with clinical relapse, while low levels are associated with a low risk of relapse in CD patients and predict treatment discontinuation [86]. In ulcerative colitis patients, only a modest increase in CRP is observed, and it is estimated that ~15% of patients in the general population may not show a CRP response due to genetic heterogeneity in CRP responses. CRP has the benefit of being widely available and inexpensive to perform, and it can be performed using the same blood specimen collected for other laboratory tests. However, it is a marker of systemic inflammatory response and thus not specific to IBD (Table 3). We recently reported that while CRP was elevated in line with mucosal inflammation on biopsies, the results were highly variable, consistent with its poor reliability in IBD as a marker of active disease [64]

#### 2.3.4. Perinuclear Anti-Neutrophil Cytoplasmic Antibody (pANCA) and Anti-*Saccharomyces cerevisiae* Antibody (ASCA)

The perinuclear anti-neutrophil cytoplasmic antibody (pANCA) is an autoantibody directed against the cytoplasmic granules of neutrophils, specifically myeloperoxidase, while anti-*Saccharomyces cerevisiae* antibodies (ASCAs) (IgA and IgG) are antibodies directed against the cell wall of the yeast *S. cerevisiae*, which shares homology with intestinal bacteria [87,88]. When used together, these two antibodies are able to distinguish between CD and UC. In CD, the characteristic antibody pattern of ASCA-positive and pANCA-negative tests had a sensitivity, specificity, and positive predictive value (PPV) of 49%, 97%, and 96%, respectively [89,90]. Positive pANCA and negative ASCA test results, on the other hand, yielded 57%, 97% and 92.5% sensitivity, specificity, and PPV, respectively, for UC. The characteristic antibody pattern can be used to discriminate between CD and UC. These two antibodies have also been detected in sera for years prior to diagnosis of IBD, suggesting an important prognostic role of these serological markers in predicting future disease [91,92] (Table 3). Furthermore, ASCA-positive CD patients have a higher risk of more severe disease, including strictures, early onset, perianal disease, and need for surgery [93,94]. Higher serum ANCA levels were reported to be associated with severe disease [90], and in pediatrics, higher ASCA titers were associated with perianal disease [95]. The gold standard methodology for determining pANCA is indirect immunofluorescence. The important limitations of indirect immunofluorescence on tissue substrates include specialized training, an expert and skilled technologist to interpret results, being time- and labour-intensive, and subjective interpretation (Table 3). The enzyme-linked immunosorbent assay (ELISA) can also be used for quantitative determination of pANCA levels and can distinguish between different ANCAs [96]. The most widely used method for determining ASCA is the ELISA, followed by immunofluorescence. Newer methods include multiplex immunoassays and immunoblotting for both antibody types.

Inflammatory bowel diseases are chronic, inflammatory disorders of the GI tract whose diagnosis and treatment monitoring rely on assessing mucosal inflammation. Fecal calprotectin has emerged as a key non-invasive biomarker that reflects neutrophil-driven gut inflammation and is used to distinguish IBD from IBS. Other biomarkers used in conjunction with FC for the assessment of disease activity include CRP and pANCA. ASCA could be used to distinguish between UC and CD and predict disease severity and localization.

### 2.4. Irritable Bowel Syndrome

Irritable bowel syndrome (IBS) is a chronic, debilitating disorder of gut–brain interactions with a variable prevalence estimated to be between 10 and 20% [97]. According to the Rome IV criteria, IBS is defined as “individuals experiencing chronic abdominal pain at least once a week in the last 3 months” [98,99]. The disease is characterized by abdominal pain, bloating, and irregular bowel movements, manifesting mainly as either constipation, diarrhea, or a mixture of constipation and diarrhea. The disease is frequently diagnosed in middle-aged individuals, with a higher incidence in females. The diagnosis of IBS is performed by excluding other organic GI disorders, such as IBD and celiac disease, using self-reported symptoms by patients, fulfilling the Rome IV criteria [98]. To exclude other organic GI disorders, fecal calprotectin or lactoferrin, stool testing for Giardia, serologic testing to exclude celiac disease, and CRP (when FC or FL are not available) are performed to exclude IBD, Giardia, and celiac disease, respectively [70,100]. In patients with IBS symptoms, a CRP level of ≤0.5 mg/L or an FC level of ≤40 μg/g is used to rule out IBD [70,100].

Serology testing for anti-cytolethal distending toxin B (anti-CdtB) and antivinculin antibodies is currently being evaluated to support the diagnosis of IBS [101,102]. However, to confirm their diagnostic role, additional studies in larger patient cohorts are still required before their clinical use. Until these tests are analytically and clinically validated and approved for clinical use, IBS will continue to be diagnosed by exclusion.

### 2.5. Colorectal Cancer

Colorectal cancer (CRC) is the third most diagnosed cancer and the second leading cause of cancer deaths worldwide [103]. In Canada and the United States of America, the incidence of CRC is higher in those over 50 years, with recent evidence showing an increasing incidence in those over 45 years [104,105,106,107,108]. The disease is more prevalent in men (10%) than in women (9.2%) worldwide. The risk factors for the development of CRC include age > 50 years, even though there was a recent increase in the diagnosis of CRC in adults between 45 and 50 years with no family history; a family history of CRC; a history of chronic diseases, such as IBD and celiac disease; and a sedentary lifestyle, obesity, poor dietary habits, smoking, and alcohol consumption [109,110]. The development of CRC is gradual, starting from alterations in the healthy colonic epithelium and progressing to the development of benign adenomatous polyps, which, over time, can become malignant and develop into cancerous lesions [111]. Polyps are premalignant lesions, and early intervention through diagnostic and therapeutic colonoscopy prevents progression into full-blown cancer and cures the disease. Many countries have established screening programs for the early detection of CRC in people at an average risk of developing CRC. Age is the single most important risk factor, with 50 years being the age to begin screening for average-risk individuals in most countries. In the most recent US preventive task force guidelines, the age to begin screening was lowered to include those between the ages of 45 and 49 years [112]. Those who screen positive undergo follow-up testing by colonoscopy, which is the gold standard diagnostic test for colon cancer. Below, we will briefly discuss the biochemical screening tests used for CRC.

The presence of blood (from the lesions) in stool is the principal biochemical biomarker used for CRC screening. There are two main tests used for detecting blood in stool—the guaiac-based fecal occult blood test (gFOBT), which detects heme, and the fecal immunochemical test (FIT), which detects the globin component of hemoglobin (Table 4). The gFOBT is a chemical test that utilizes the pseudoperoxidase activity of heme (in hemoglobin) to detect the presence of blood in stool samples. The FIT, on the other hand, is an immunoassay that utilizes antibodies against human globin to detect the globin component of hemoglobin in stool. The major advantages of the FIT over the gFOBT are summarized in Table 4 [113,114,115,116]

Another stool test recently approved by the United States Food and Drug Administration (FDA) for CRC screening is the Cologuard^®^ test. It is a molecular multitarget stool DNA test (mts-DNA) targeting aberrant DNA associated with CRC and precancerous lesions, including seven mutations on the *KRAS* gene, the FIT, aberrantly methylated BMP3 and NDRG4, and human actin for normalization [117]. In terms of performance, the test has a higher sensitivity for detecting stages 1–4 colon cancer (92–94%) but lower specificity (87%) compared to the FIT [118]. Another limitation of the mts-DNA test is the complexity of sample collection, requiring a special buffer in a large jar [116,119].

Methylated septin 9 (mSEPT9) is the only blood-based CRC detection test currently approved by the FDA for CRC screening. Its use is limited to individuals who decline or are unable to complete higher-efficacy screening tests [116,120,121]. The test is, however, not included in any guideline to date due to its inferior sensitivity and specificity compared to the FIT.

Colorectal cancer (CRC) is one of the most commonly diagnosed cancers and a leading cause of cancer deaths worldwide, with rising incidence among adults aged 45–49 years. Screening programs primarily use stool-based tests to detect occult blood, with the FIT offering superior sensitivity, specificity, and convenience compared with the gFOBT. Additional tests, such as multitarget stool DNA (Cologuard^®^) and blood-based mSEPT9, are available, but the FIT remains the most effective and widely recommended screening tool, followed by confirmatory colonoscopy.

## 3. Emerging Laboratory Tests and Biomarkers in GI Diseases

### 3.1. Gut Microbiota as a Diagnostic Tool in IBD

The intestinal microbiota has been suggested to play an important role in the pathophysiology of IBD, including UC and CD, with differential microbial diversity, composition, and abundance in IBD patients compared to controls [122]. For example, some studies have reported a relatively high concentration of mucosal bacteria in patients with IBD, with bacteria concentrations correlating with disease severity [123]. Patients with IBD have been reported to have a higher abundance of *Proteobacteria* and *Actinobacteria* and lower amounts of *Bacteroides*, *Eubacterium*, and *Faecalibacterium* than healthy individuals [124,125,126]. These differences in composition, abundance, and diversity have been exploited to develop a potential laboratory diagnostic test for the diagnosis of IBD. Zheng et al. developed a multiplex droplet digital polymerase chain reaction (m-ddPCR)-based multibacteria biomarker panel to quantify selected species enriched or depleted in IBD patients [127]. This multibacteria biomarker panel showed better performance than FC in UC, with comparable performance to FC in the CD model. In CD, the multibacteria biomarker panel was able to differentiate patients with active and inactive disease from controls and showed higher performance than FC in discriminating inactive UC from controls [127]. The strengths of their study include the diversity of the IBD cohort population (across multiple countries) and the strong clinical performance of the test in distinguishing between IBD and non-IBD patients across all the populations studied. The control subjects were all recruited from a single center, and no record of their diet was taken, which is known to impact the gut microbiota. Bacterial detection based on m-ddPCR is more user-friendly and relatively cheaper. with less variability in results between operators. Robust clinical evaluations, including studies in different populations to confirm the analytical and clinical performance of the multibacteria biomarker panel compared to the current standard of care tests, are required prior to clinical implementation.

A fecal whole-metagenome shotgun sequencing and machine learning predictive model was also used to develop a potential diagnostic tool to distinguish between UC and CD in IBD patients. This model uses the differential abundance and diversity of gut microbiota. Using this model, the abundances of *Alistipes shahii* and *Pseudodesulfovibrio aespoeensis* were negatively correlated with the severity of CD, whereas the abundance of *Polynucleobacter wianus* was positively correlated. The severity of UC was negatively correlated with the abundance of *A. shahii*, *Porphyromonas asaccharolytica*, and *Akkermansia muciniphilla*, while it was positively correlated with the abundance of *Pantoea candidatus pantoea carbekii* [128]. It is important to note that this model combined genome sequencing and machine learning for prediction. The sample size was relatively small (80 subjects), with no diversity in the population to draw conclusions using a machine learning model, and cofounders that may affect the gut microbiome, such as age, body mass index, and lifestyle, were not accounted for.

Gut microbiota-based biomarkers, such as multibacteria panels, metagenomic sequencing, and machine learning models, can differentiate between IBD subtypes, track active versus inactive disease, and correlate specific bacterial abundances with disease severity. Combining currently used tests, such as FC and CRP, with microbiota profiles offers a complementary approach, where FC provides a rapid, non-invasive measure of inflammation, and microbiota data adds diagnostic specificity and predictive information. This integrated strategy can enhance patient stratification, guide therapy decisions, and potentially identify early dysbiosis leading to disease.

While the microbiota may have utility in distinguishing between IBD patients and normal controls and between CD and UC, its use in the clinical laboratory will require clinical validation, easy-to-use and cost-effective instrumentation, and minimum manual sample preparation for effective uptake.

### 3.2. Gut Microbiome-Associated Serum Metabolites (GMSMs) in IBD and CRC

The altered composition, diversity, and abundance of the gut microbiota in IBD result in altered microbial metabolic pathways and, consequently, differential expression of microbial metabolites, which can be used as a diagnostic tool. Pathways involved in amine degradation and lipid biosynthesis were significantly enriched in patients with IBD [129]. Pathways belonging to amino acid biosynthesis contributed by bacterial species depleted in patients with UC, including *C. leptum*, *F. saccharivorans*, *G. formicilis*, and *R. torques*, are associated with a significant decrease in the abundance of their respective metabolites [127]. Serum levels of the amino acid tryptophan were significantly lower in patients with IBD than in controls, with a stronger reduction in patients with CD than in patients with UC [130]. The same study also reported a negative correlation between serum tryptophan levels and disease activity or CRP levels, as well as a differential fecal microbiota composition associated with tryptophan metabolism [130]. Furthermore, following successful clinical intervention with infliximab, tryptophan levels showed a sustained increase, consistent with the role of tryptophan as a predictor of clinical improvement. Thus, serum tryptophan and its metabolites might serve as useful biomarkers for IBD; however, further studies are required to characterize an appropriate clinical threshold for low tryptophan.

The composition and abundance of the gut microbiota have also been shown to be significantly altered in patients with adenoma or CRC, with increases in *Bacteroides*, *Parvimonas*, *Bilophila*, and *Fusobacterium*, and decreases in *Ruminococcus*, *Bifidobacterium*, and *Streptococcus* species [131,132,133]. This, in turn, results in altered metabolic pathways in the gut environment, resulting in the generation of differential microbial-associated metabolites. Measurements of these metabolites in serum can be used to discriminate between CRC patients and controls. Chen et al. described a gut microbiome-associated serum metabolite (GMSM) panel, including short-chain fatty acids, polyamines, phenols, indole derivatives, spermidine, and unconjugated secondary bile acids, measured by liquid chromatography–mass spectrometry. The panel was used to accurately discriminate CRC and adenoma samples from normal samples, with a sensitivity and specificity of 83.5% and 84.9%, respectively [133,134]. With the rapid advancement in analytical methodologies, measurements of GMSM could be combined with other markers of CRC to facilitate the early detection of CRC and adenomas.

Gut microbiota-associated serum metabolites may be used to provide insights into disease-specific metabolic and microbial changes that drive inflammation, with metabolite panels potentially utilized to improve disease specificity, differentiate between CD and UC, and detect early changes in CRC or adenomas. The use of these biomarkers in conjunction with currently available tests can provide a multi-dimensional approach to diagnosis, monitoring, and personalized management, enhancing early detection and patient stratification. Their clinical use will require clinical validation in larger and diverse patient populations.

### 3.3. Other Potential Biomarkers in IBD

#### 3.3.1. Anti-Integrin αvβ6 Autoantibodies

Integrins are cell surface heterodimeric glycoproteins consisting of α and β subunits involved in cell signalling, proliferation, cell adhesion, and migration. They are thought to play a central role in the pathogenesis of UC, with clinical evidence of blocking integrin and preventing integrin-mediated intestinal trafficking of lymphocytes [134,135]. Integrin αvβ6 has been reported to play a part in maintaining epithelial barrier functions [136,137]. Recently, anti-integrin αvβ6 autoantibodies have been reported to be elevated in individuals who develop UC and precede the development of clinical disease by about 10 years [138]. Furthermore, it has been reported that there is a correlation between anti-integrin αvβ6 autoantibodies and disease severity in UC patients [139,140]. These studies highlight the potential utility of anti-integrin αvβ6 autoantibodies as both a prognostic and diagnostic marker of IBD, specifically UC. In these studies, serum samples were used to determine anti-integrin αvβ6 autoantibody levels, which may increase compliance in patients. The use of serum samples for this test is an attractive option, as it will increase compliance among IBD patients; however, more clinical studies are required in larger and diverse patient populations, as well as in newly diagnosed IBD patients, to optimize an appropriate clinical cut-off.

#### 3.3.2. Oncostatin

Recently, plasma oncostatin, a member of the interleukin-6 (IL-6) cytokine family whose expression in the intestinal stroma was shown to be correlated with the presence and severity of inflammation, has emerged as a potential prognostic marker of disease remission in IBD patients undergoing anti-tumour necrosis antagonist (anti-TNF) therapy [141,142]. Elevated plasma oncostatin levels are associated with poor therapeutic response to anti-TNF therapy in IBD patients, with high pretreatment oncostatin levels capable of identifying anti-TNF therapy non-responders [142]. Further assay optimization is required to standardize an oncostatin concentration cut-off that can discriminate between responders and non-responders to anti-TNF therapy.

Plasma oncostatin can thus be used on its own or to complement current laboratory tests for IBD, like fecal calprotectin and CRP, by providing prognostic information on the therapeutic response. While FC and CRP reflect current inflammation, elevated pretreatment oncostatin levels can identify patients who are unlikely to respond to anti-TNF therapy. Combining these markers enables more personalized treatment decisions, optimizing therapy selection and disease management.

#### 3.3.3. MicroRNA (miRNA) as a Potential Biomarker of IBD

The differential expression of microRNAs (miRNAs) in IBD and CRC is also being examined as a potential non-invasive molecular diagnostic tool for both GI diseases. In IBD, miR-192-5p, let-7b-5p, miR-495-5p, miR-361-3p, and miR-124-3p, among others, have been reported to be differentially expressed in patients with IBD compared to normal patients [143]. miRNAs may have an important prognostic and diagnostic role in GI disorders. Advancement in this area is still early and should be revisited in the future.

Serum anti-integrin αvβ6 autoantibodies and plasma oncostatin are important emerging biomarkers in IBD. They can predict disease onset, correlate with disease severity, and identify patients unlikely to respond to anti-TNF therapy. The differential expression of miRNAs holds potential as non-invasive diagnostic and prognostic markers, but further studies are required.

### 3.4. Interleukin 2 (IL-2) for the Diagnosis of Celiac Disease

A new whole blood assay measuring IL-2 (WBAIL-2) with potential to aid in the diagnosis of celiac disease has been described [144]. Gluten-specific CD4+ T cells with specificity to celiac disease play a central role in the pathogenesis of celiac disease and can be detected by functional T cell assays. Alternatively, humoral immune products triggered by these CD4+ T cells, including cytokines IL-2, IL-8, and IL-10, can also be measured. IL-2 has been shown to have high sensitivity and specificity in distinguishing celiac disease patients from healthy controls, as well as non-celiac gluten sensitivity. In the WBAIL-2 test, a mixture of eight purified synthetic immunoreactive gluten stimulatory peptides (DQ2.5-glia-α1b, DQ2.5-glia-α2, DQ2.5-glia-ω1, and DQ2.5-glia-ω2 epitopes, as well as HLADQ2.5, HLA-DQ2.2, and HLA-DQ8—restricted epitopes derived from wheat α-, γ-, and ω-gliadins and low-molecular-weight glutenin) are added to whole blood collected from patients and incubated for 24 h at 37 °C. After incubation, plasma is separated and used for the determination of IL-2. The IL-2 assay demonstrated high accuracy for celiac disease diagnosis, even in patients consuming a strict gluten-free diet, with a sensitivity and specificity of 90% and 95%, respectively, for HLA-DQ2.5+ patients. This test is not yet available for clinical use. Current serology tests require patients to be on a normal gluten diet prior to testing, which is very inconvenient for patients. This test will obviate the need for a gluten diet before testing and may also have clinical utility in seronegative celiac patients.

### 3.5. Gluten Immunogenic Peptide (GIP) for Diet Compliance and Unintentional Gluten Exposure

Gluten immunogenic peptides (GIPs) are gluten components resistant to gastrointestinal digestion and are excreted in urine or stool [145]. Glutens are the protein complexes implicated in the pathogenesis of celiac disease. Gluten immunogenic peptide (GIP) can be used to assess compliance with a gluten-free diet and to detect unintentional exposures [146]. Several studies have reported that GIP testing can detect gluten exposure missed by traditional monitoring tools [45,145]. Controlled gluten-challenge trials have demonstrated that stool GIP assays are highly sensitive even at low gluten doses and outperform urine GIP, serology, and adherence scores in detecting intermittent gluten exposure [146]. However, some pediatric studies reported that GIP detection did not always correlate with self-reported adherence, serology, or symptoms, highlighting potential challenges in its clinical use [147]. Gluten immunogenic peptide testing can thus provide an objective tool to assess diet adherence and exposure to gluten.

## 4. Conclusions

GI diseases are responsible for a significant and expanding burden of health care utilization and expenses. Understanding the various methodologies, along with their strengths and limitations, is important for laboratory professionals and clinicians to meet the burden of various GI diseases. A combination of endoscopy and histology with non-invasive fecal- and blood-based biomarkers is incorporated into many clinical guidelines for screening, diagnosis, and therapeutic monitoring of most GI diseases. There is now emerging interest in developing novel non-invasive diagnostic tools for GI diseases. The role of the gut microbiota in the pathophysiology of GI disorders is being deciphered, and the gut microbiome and its related serum metabolites have been identified as potential diagnostic and prognostic targets. New prognostic tests are being developed, especially those that can be used to predict the response to therapy or disease progression. While the number of novel biomarkers is expanding, and they appear to be useful in discriminating between healthy controls and various GI diseases, none are routinely used for clinical diagnosis at this time. These biomarkers may be used either independently or in combination with current biomarkers; however, further clinical validation of these markers in larger and more diverse populations, as well as increasing the availability of easy-to-use instrumentation and minimal and standardized sample preparation, is required before their incorporation into routine clinical use and inclusion in diagnostic algorithms.

## Figures and Tables

**Table 1:** Clinical utility, advantages, and limitations of different *H. pylori* tests.

Laboratory Test	Clinical Utility	Advantages	Limitations
Urea breath test (UBT)	Detection of active *H. pylori* infection, disease monitoring, and effectiveness of treatment.	High sensitivity and specificity. Detects active infection. Can be used to confirm cure.	Requires consumption of a urea drink. Inconvenient for some patients, not recommended for children, and false negatives due to recent PPIs, antibiotics, bismuth, and histamine H1 inhibitor. Compliance may be challenging in some groups of patients. Requires specialized instruments and training.
*H. pylori* stool antigen test (HpSAT)	Detection of active *H. pylori* infection, disease monitoring, and effectiveness of treatment.	High sensitivity and specificity. Detects active infection; confirms cure. Available in automated analyzers and easy to perform. Recommended for use in children and pregnant women. Convenient for drop off stool at any time.	Requires collection of a stool sample, which may be inconvenient for some patients. Variability in results due to sampling. Requires proper sample handling. False negative may occur due to recent therapy. Stool consistency may affect results.
Serology	Detection of *H. pylori* infection.	Highly sensitive and specific. Widely available and easy to perform. Cost-effective and inexpensive. No requirement to stop antibiotics, PPIs, or bismuth.	Cannot be used to distinguish between active and previous infection. Low specificity in regions of low prevalence. Cannot confirm cure (antibodies are persistent).
*H. pylori* molecular tests	Detection of *H. pylori* infection and antibiotic resistance.	Highly sensitive and specific. Capable of detecting very low bacterial loads. Capable of identifying antibiotic (clarithromycin) resistance mutations. Can be used to confirm cure.	Requires specialized equipment. Expensive compared to the HpSAT and UBT. May detect DNA from dead bacteria, leading to false positive results. False negatives may occur due to inhibitors of PCR in fecal samples.

**Table 2:** Clinical utility, advantages, and limitations of different celiac disease tests.

Laboratory Test	Clinical Utility	Advantages	Limitations
Anti-tTG IgA serology	First-line screening test for celiac disease.	Highly sensitive and specific. Widely available and cost-effective. High concentrations can be used to confirm a diagnosis of celiac disease.	Patients must be on a normal gluten diet before testing. False negatives in IgA deficiency (requires concomitant performance of IgA). False negatives may occur in children <2 years.
EMA-IgA/IgG Serology	Second-line screening test and confirmatory test.	Highly specific and, together with a positive anti-tTG IgA/IgG, can be used to confirm a diagnosis of celiac. Detection of both EMA-IgA and -IgG can be useful in IgA-deficient cases.	Lower sensitivity compared to anti-tTG IgA. Very labour-intensive (immunofluorescence on monkey esophagus). Subjective interpretation in manual microscopy. Requires specialized microscopy and training. Expensive. Patients must be on a normal gluten diet before testing.
DGP-IgG	Useful in children <2 years old and in IgA deficiency.	Can detect some cases missed with anti-tTG.	Lower specificity in adults.
HLA-DQ2/DQ8	Used as a rule-out test for celiac (negative results, rules out celiac disease).	Very high negative predictive values. A gluten diet is not required.	Low positive predictive value (positive HLA-DQ2/DQ8). Does not confirm disease.

**Table 3:** Clinical utility, advantages, and limitations of the different tests used to assess inflammation in IBD.

Laboratory Test	Clinical Utility	Advantages	Limitations
Fecal calprotectin	Helps to differentiate IBD from IBS, assess mucosal healing, where concentrations reflect active inflammation, monitor therapy, and predict relapse.	Highly sensitive and specific to intestinal inflammation. Can be used to triage patients for endoscopy. Cheap, non-invasive test. Available in automated laboratory analyzers.	Requires the collection of stool samples; as such, it is inconvenient to some patients. Sensitive to intestinal inflammation but not specific to IBD (see Figure 2). Variability in results due to sampling issues. Some methodologies require manual sample preparation.
CRP	Levels correlate with disease activity and active inflammation; levels correlate with clinical relapse and can be used to predict treatment discontinuation.	Cheap, widely available, and can be performed using a small blood sample.	Systemic marker of inflammation not specific to IBD. Due to genetic heterogeneity, some patients may not show a CRP response.
pANCA	Used in combination with ASCA to discriminate between UC and CD. Positive pANCA and negative ASCA specific for UC; helps to distinguish between UC and CD. Antibody levels correlate with disease severity.	High specificity and PPV for UC. When used together with ASCA, it can be used to discriminate between UC and CD. Serum test; non-invasive test.	Low sensitivity and accuracy for UC. Testing by immunofluorescence is expensive, labour-intensive, and requires a fluorescent microscope; interpretation is subjective.
ASCA	When used in together with pANCA, positive ASCA is specific to CD and can be used to distinguish between CD and UC and predict disease. Antibody levels correlate with disease localization in pediatrics.	High specificity and PPV for CD. When used together with pANCA, it can be used to discriminate between CD and UC.	Low sensitivity and accuracy for CD.

**Table 4:** Clinical utility, advantages, and limitations of the different tests used for CRC screening.

Laboratory Test	Clinical Utility	Advantages	Limitations
FOBT	Screening test for CRC.	Highly cost-effective. Widely available. No specialized instrumentation required. Easy to perform.	Vulnerable to interferences, which could result in false positive (e.g., from meat, beet, oxidizing agents) or false negative (some medications, vitamin C, antioxidants) results. The test cut-off is determined by the manufacturer and cannot be adjusted. Provides a qualitative result only. Requires three different specimens to complete testing.
FIT	Screening test for CRC.	Immunoassay-based, providing greater analytical specificity and sensitivity. Cost-effective. Can be automated. Less vulnerable to interference from diet and medications. Provides quantitative value with an adjustable cut-off. Requires only a single specimen.	Requires specialized instruments for testing.
mts-DNA (Cologuard)	Screening for CRC.	High sensitivity.	Lower specificity compared to the FIT. Complex sample collection Requires specialized instrumentation and training.

## Data Availability

No new data were created or analyzed in this study. Data sharing is not applicable to this article.
